# The impact of aggregating serogroups in dynamic models of *Neisseria meningitidis* transmission

**DOI:** 10.1186/s12879-015-1015-8

**Published:** 2015-07-30

**Authors:** Keith D Poore, Chris T Bauch

**Affiliations:** Department of Mathematics and Statistics, University of Guelph, 50 Stone Road East, Guelph, ON Canada; Department of Applied Mathematics, University of Waterloo, 200 University Avenue West, Waterloo, ON Canada

## Abstract

**Background:**

*Neisseria meningitidis* (*Nm*) is a pathogen of multiple serogroups that is highly prevalent in many populations. Serogroups associated with invasive meningococcal disease (IMD) in Canada, for example, include A, B, C, W-135, X and Y. IMD is a rare but serious outcome of *Nm* infection, and can be prevented with vaccines that target certain serogroups. This has stimulated the development of dynamic models to evaluate vaccine impact. However, these models typically aggregate the various *Nm* serogroups into a small number of combined groups, instead of modelling each serogroup individually. The impact of aggregation on dynamic *Nm* model predictions is poorly understood. Our objective was to explore the impact of aggregation on dynamic model predictions.

**Methods:**

We developed two age-structured agent-based models--a 2-strain model and a 4-strain model--to simulate vaccination programs in the Canadian setting. The 2-strain model was used to explore two different groupings: C, versus all other serogroups combined; and B, versus all other serogroups combined. The 4-strain model used the four groupings: C, B, *Neisseria lactamica*, versus all other serogroups combined. We compared the predicted impact of monovalent C vaccine, quadrivalent ACWY vaccine (MCV-4), and monovalent B vaccine (4CMenB) on the prevalence of serogroup carriage under these different models.

**Results:**

The 2-strain and 4-strain models predicted similar overall impacts of vaccines on carriage prevalence, especially with respect to the vaccine-targeted serogroups. However, there were some significant quantitative and qualitative differences. Declines in vaccine-targeted serogroups were more rapid in the 2-strain model than the 4-strain model, for both the C and the 4CMenB vaccines. Sustained oscillations, and evidence for multiple attractors (i.e., different types of dynamics for the same model parameters but different initial conditions), occurred in the 4-strain model but not the 2-strain model. Strain replacement was also more pronounced in the 4-strain model, on account of the 4-strain model spreading prevalence more thinly across groups and thus enhancing competitive interactions.

**Conclusions:**

Simplifying assumptions like aggregation of serogroups can have significant impacts on dynamic model predictions. Modellers should carefully weigh the advantages and disadvantages of aggregation when formulating models for multi-strain pathogens.

**Electronic supplementary material:**

The online version of this article (doi:10.1186/s12879-015-1015-8) contains supplementary material, which is available to authorized users.

## Background

*Neisseria meningitidis* (*Nm*) is a significant concern to public health. Prevalence of carriage varies between 5 and 10 % of the population [1]. Six main serogroups impact public health: A, B, C, W-135, X, and Y [1]. In Canada, serogroup B is most prevalent, although C, X, and Y are part of the ecological landscape as well, and are targeted by immunization programs at the time of writing [2–4]. Incidence of invasive meningococcal disease (IMD) in Canada ranges from 0.4 to 0.7 per 100,000 individuals [2], which is small compared to the disease burden in some countries, such as the “meningitis belt” of sub-Saharan Africa.

*Nm* infection is usually commensal: the pathogen typically colonizes the mucosal layer of the nasopharynx asymptomatically. *Nm* can be spread through the aerosol route, including coughing and sneezing, but also through close contact [2]. Therefore, closed environments, such as schools, households, and workplaces yield conditions conducive to transmission [5–10]. IMD incidence tends to be highest in children under 2 years of age, while *Nm* carriage tends to be highest in adolescent teenagers and young adults (17–21 years of age).

IMD symptoms develop when the colony penetrates the mucosal layer into the bloodstream, leading eventually to meningitis septicaemia [11]. Targeting the meninges and causing IMD, *N. meningitidis* can cause permanent neurological damage or fatality if not treated promptly. Many who survive IMD suffer loss of limbs or hearing. Determinants of individual susceptibility to IMD are not well understood.

There are several routes to natural immunity to *Neisseria meningitis*. After being infected with any serogroup of *Nm*, many individuals produce anti-bodies that prevent invasion by all serogroups of the pathogen, reducing the risk of becoming infected, although the duration of immunity is unknown, and probably relatively short [12]. Another route to transient *Nm* immunity is contracting *Neisseria lactamica* (*Nl*), a commensal pathogen. Though it is known that *Nl* colonizes the same loci as *N. meningitidis*, the mechanism and nature of the immunity is under study and currently not well known, though ideas of creating a vaccine using *Nl* have been proposed [13, 14]. *N. lactamica* colonization induces Immunoglobin A and Immunoglobin G antibody production, preventing recolonization for at least 24 weeks [15]. Though the mechanism is not clear, it is known that an immunological response to *Nl* causes an immunological response to *Nm*.

*N. meningitidis* and *N. lactamica* compete with one another for the same loci in the nasopharynx, as do the various N. meningitidis serogroups. If one pathogen is introduced to a host while the other has already colonized, the invading pathogen will not easily colonize due to competition for nutrients or adhesion [13].

Currently, vaccines reduce carriage and IMD rates in vaccinated individuals, and also unvaccinated individuals through herd immunity [16]. Previous polysaccharide vaccines were relatively ineffective, and could cause side effects [17]. However, in 2001 a conjugate vaccine that targets serogroup C was recommended for use in Canada for infants and children [18]. IMD incidence attributable to serogroup C was greatly reduced amongst the age groups that were vaccinated, as well as those that were not vaccinated [18]. However, with the reduced incidence of IMD attributable to serogroup C (and presumably, carriage as well), a small gap has been left in the ecological niche: there is less asymptomatic carriage of serogroup C in the nasopharyx, which, in principle, means more opportunities for other serogroups to colonize. This has caused some concern over potential vaccine-induced strain replacement [19]. We speculate that this ‘ecological’ strain replacement should be even stronger for vaccines against very common serogroups, such as serogroup B, if serogroups compete for space in the nasopharynx.

In 2007, Canada’s National Advisory Committee on Immunization recommended a quadrivalent conjugate *Nm* vaccine, meningococcal conjugate vaccine (MCV-4), that immunizes against serogroups A, C, W-135, and Y. A routine vaccine program was recommended for adolescent individuals in areas that local epidemiology warranted the prevention of the outbreaks, while high risk individuals were also recommended for vaccination [20].

Serogroup B causes significant IMD in many countries, since it dominates the ecological landscape of its environment [21]. Vaccines against serogroup B have been difficult to produce because the capsular polysaccharides for serogroup B are too similar to human neural antigens [22, 23]. Previous attempts required up to 20 different types of the antigen, PorA [24]. Though there have been difficulties, a vaccine has been developed using only four different components, 4CMenB [25]. With the use of 4CMenB in addition to MCV-4, it has been predicted that more than three quarters of the various *Nm* serogroups in Europe can be covered [26]. It is also predicted that 4CMenB can be used to target certain strains of serogroup X, which no vaccine is currently able to cover [27]. 4CMenB is one step closer to a universal vaccine for *Nm*, since the components found in the vaccine can also be found in all other capsule groups, thus 4CMenB may have an impact on other serogroups, although perhaps less efficaciously than the MCV-4 vaccine.

Previous models describing the impact of immunization on population health have aggregated all *Nm* serogroups into only two or three groups [28–31]. Some models aggregated all serogroups of *Nm* while modeling the effects of *Nl* [28]. Other models have analyzed a single vaccine applied to different age categories [2, 30, 31], while some models compare vaccine types [2]. These models can produce valuable insights, and aggregating serogroups is a necessary model simplification. However, aggregating serogroups can also influence model projections because the ecological effects of serogroup structure and serogroup interactions cannot be fully accounted for [32, 33], and it is known that multi-strain models can produce qualitatively different dynamics from single-strain models [34–38]. To our knowledge no research has directly compared the results of few-strain versus many-strain dynamic *Nm* models.

Here we develop, analyse and compare two age-structured, agent-based transmission models. The first model aggregates all serogroups into one of two categories while the second model aggregates all serogroups into one of four categories. Our objective is to compare the predicted impacts of monovalent C, monovalent B, and quadrivalent CAWY vaccines on *Nm* carriage using the two models, in order to learn more about potential biases introduced by using few-strain models to model multi-strain infectious diseases such as *Nm*.

## Methods

### 4-strain model

Our 4-strain model utilizes four groupings: (1) serogroup B, (2) serogroup C, (3) serogroups A, W-135, X, Y, 29e, and ‘ungroupable’ (UG) serogroups, and (4) *Neisseria lactamica*. We include *Neisseria lactamica* since it competes for the same loci on the nasopharynx as *Neisseria meningitidis* serogroups [13]. Serogroups A, W-135, X, and Y are rare in Canada but 29e and the ungroupable serogroups are more prevalent. Hence, in the 4-strain model, serogroup B, serogroup C, and *Nl* form their own compartments, whereas other serogroups are lumped into the same compartment with one or more other serogroups.

The population is divided into annual age cohorts: <1 year old, 1 year olds, 2 year olds, 3 year olds, …, 99 year olds. Individual ages are updated monthly, moving the individual into the next age cohort after 12 simulated months. Individuals are removed each month according to all-cause, age-specific mortality rates [39] (Table S1 in Additional file 1). Individuals are born into the population at a constant rate, η, each month. A fine age structure enables comparing scenarios that distinguish between vaccinating at 12 months versus 24 months, for instance. However, for model calibration, broader age categories of 0–4, 5–9, 10–14, 15–19, and 20+ years of age were used, because 5-year age intervals are used in most available *Nm* carriage prevalence data as well as in the available contact data [40].

Disease transmission includes a baseline transmission rate specific to each age group and strain, β_i,k_, that is modified by an age specific contact matrix, C_i,j_ [40]. The contact matrix includes both the physical and non-physical contact of individuals as *Nm* is transmitted through aerosol means. Newborns are susceptible to all serogroups of *Nm* and to *Nl*. The probability per timestep that an individual of age j is infected by serogroup k is given by

1 $$ p\left({I}_{i,k}\right)=1-{\displaystyle {\prod}_{j=0}^{100}\left(1-\left(\frac{\beta_{i,k}{C}_{i,j}{I}_{j,k}}{N_j}\right)\right)} $$

where *I*_*i,k*_,is the number of individuals of age *i* infected with strain *k* (carrying the strain asymptomatically), and *N*_*j*_ is the number of individuals of age, *j*. Upon infection, individuals become a carrier of the *k*^*th*^ serogroup.

No individual can be infected with more than one serogroup simultaneously. After the duration of carriage, *τ*_*1k*_, has elapsed the individual becomes naturally immune to serogroup *k* that infected them, for a short duration *τ*_*2k*_ sampled from a gamma distribution. We assume that the average duration of carriage is the same for all ages and *Nm* serogroups. The average duration of carriage for *Nl* is also assumed to be the same for all ages, but differs from the average duration of carriage of *Nm* [13, 41]. While natural immunity to serogroup *k*, individuals also have some partial cross-protection against other serogroups, so that the probability per timestep that an individual of age *j* is infected by some other serogroup *k* is given by

2 $$ p\left({I}_{i,k}\right)=1-{\displaystyle {\prod}_{i=0}^{100}\left(1-\left(\frac{\beta_{i,k}{C}_{i,j}{I}_{j,k}}{N_j}\right)\left(1-{\sigma}_l\right)\right)} $$

where, 0 < σ_l_ <1 is the cross-immunity conferred by previous infection by serogroup l ≠ k. Cross-immunity lasts the same period of time as strain-specific immunity. Details on all parameter values appear in Additional file 1: Table S1.

### 2-strain model

The 2-strain model aggregates *Nl* and *Nm* serogroups, but it uses two groupings instead of four. The first version of the 2-strain model uses the groups: C versus *Nl* and all other *Nm* serogroups aggregated (A/B/W-135/X/Y/29e/UG/*Nl*). The second version uses the groups B versus *Nl* and all other *Nm* serogroups aggregated (A/C/W-135/X/Y/29e/UG/*Nl*). The first version is used to simulate the impact of monovalent C vaccine and compare it to the simulated impact of the monovalent C vaccine using the 4-strain model. Likewise, the second version is used to simulate the impact of monovalent B vaccine and compare it to the simulated impact using the 4-strain model. The 2-strain model is otherwise identical to the 4-strain model. In both versions, the duration of carriage and immunity was assumed to be the duration of *Nm* and was set by sampling from a gamma distribution.

### Parameterization and uncertainty analysis

We used Canadian demographic and epidemiologic data to parameterize the model. The baseline natural history and demographic parameters were taken directly from the literature, while the baseline transmission rates β_i,k_ were calibrated so that the age-stratified serogroup-specific carriage prevalence in the model matched the empirical data, within specified ranges of acceptability (Figure S1 in Additional file 1 and Table S2 in Additional file 1 for the 4-strain model) [2, 3]. In particular, initial values of β_i,k_ were assumed for each simulation; every 100 simulated years, the code checked whether the modelled age-specific carriage prevalence was within the range of empirical acceptability based on seroprevalence data, for all of the ranges (20 for the 4-strain model, 10 for the 2-strain model); if so, those calibrated values of β_i,k_ were used in the simulation, and if not, the values of β_i,k_ were adjusted upward or downward as appropriate and the process was repeated for another 100 simulation years until the target ranges were attained.

The degree of cross-protection is not well known, so we used values from an in-vitro cross-reactivity study [42] (see Table S1 in Additional file 1 for baseline parameter values; see Tables S2, S3, S4 for the average β_i,k_ values in Additional file 1). 50 realizations for each vaccine scenario were thereby produced, each through this calibration procedure. 50 realizations were used because this was found to be a sufficient number of realizations to produce representative averages of the (stochastic) agent-based model, at baseline parameter values. All results reported are the averages and standard deviations of the 50 realizations except where otherwise noted.

The calibration targets for the 4-strain model were obtained by adding the carriage prevalence of the constituent serogroups (Table S5 in Additional file 1). Similarly, the calibration of the 2-strain model, the acceptability ranges for prevalence of carriage were different for each age group and serogroup (Tables S6, S7 in Additional file 1).

In the case of the 4-strain model, a significant number of parameter sets yielded dynamics where serogroup prevalence oscillated over time, indicating the likely presence of multiple attractors. These parameter sets were not included in the baseline analysis, but are discussed separately at the end of the Results section.

### Vaccine scenarios and assumptions

We explored nine vaccination scenarios with the 4-strain model (Table 1) and six scenarios with the 2-strain model (Table 2). Vaccination begins at t = 150 years (which provides enough “burn-in” to discard transients) and is continued for 75 years (t_end_ = 225 years).

**Table 1:** Vaccine Scenarios for the 4-strain model

4-strain Vaccine Scenarios				
	Age of Immunization	Vaccine Type	Efficacy	Coverage
1	12 months	C	97 %	90 %
2	12 years	C	97 %	80 %
3	12 months and 12 years	C	97 %	90 %/80 %
4	12 months	MCV-4	97 %	90 %
5	12 years	MCV-4	97 %	80 %
6	12 months and 12 years	MCV-4	97 %	90 %/80 %
7	12 months	4CMenB	64 %	90 %
8	12 years	4CMenB	64 %	80 %
9	12 months and 12 years	4CMenB	64 %	90 %/80 %

The efficacy of the conjugate C vaccine and the MCV-4 vaccine are assumed to be 97 % [41, 46]. The 4CMenB vaccine efficacy is assumed to be 64 % based on [44]. The duration of vaccine protection is assumed to be 4 years

**Table 2:** Vaccine scenarios for the 2-strain model

2-strain Vaccine Scenarios				
	Age of Immunization	Vaccine Type	Efficacy	Coverage
10	12 months	C	97 %	90 %
11	12 years	C	97 %	80 %
12	12 months and 12 years	C	97 %	90 %/80 %
13	12 months	4CMenB	64 %	90 %
14	12 years	4CMenB	64 %	80 %
15	12 months and 12 years	4CMenB	64 %	90 %/80 %

The efficacy of the conjugate C vaccine is 97 % [46]. The 4CMenB vaccine efficacy is assumed to be 64 % based on [44]. The duration of vaccine protection is assumed to be 4 years

For the 4-strain model, scenarios 1**–**3 use a monovalent C vaccine, scenarios 4**–**6 use a quadrivalent ACWY vaccine (MCV-4), and scenarios 7**–**9 use a monovalent B vaccine (4CMenB). For the 2-strain model, scenarios 1**–**3 use a monovalent C vaccine and scenarios 4**–**6 use the 4CMenB. Coverage of toddlers and infants was assumed to be 90 % and coverage for adolescents was assumed to be 80 %, based on Quebec coverage rates [38, 43]. The conjugate C and the MCV-4 vaccines were assumed to be 97 % efficacious in protecting inoculated individuals, while the B vaccine was assumed to be 64 % efficacious [44].

Vaccination operates in an “all-or-none” fashion, such that individuals who are efficaciously vaccinated receive full protection from infection and cannot transmit infection to others, while individuals who are not efficaciously vaccinated receive no protection and remain susceptible. Individuals are only protected against serogroups included in the vaccine (i.e., none of the vaccines, including 4CMenB, confer cross-immunity). Individuals are randomly selected each month to be vaccinated according to the vaccine coverage for their age group, and each vaccinated person is protected with a probability equal to the vaccine efficacy.

## Results

The overall prevalence of carriage decreases with the introduction of vaccination, regardless of the serogroup targeted by the vaccine or the age of immunization (Tables 3, 4, 5). Herd immunity was also observed for every vaccine scenario, since prevalence declined in age groups not targeted by the vaccine. We compare the 2-strain and 4-strain model predictions for each vaccine type in the following subsections.

**Table 3:** Reduction in prevalence of carriage with the Conjugate C vaccine

Vaccine Program	Model Type	Serogroup	t = 149	t = 160	t = 175	t = 190	Relative Change
1	2-strain model	C	0.005	0.003	0.000	0.000	−0.760
			(0.012)	(0.007)	(0.000)	(0.000)	(0.427)
		Other	0.109	0.108	0.107	0.108	−0.009
			(0.008)	(0.008)	(0.010)	(0.009)	(0.054)
		Total	0.115	0.111	0.107	0.108	−0.050
			(0.020)	(0.015)	(0.011)	(0.009)	(0.068)
	4-strain model	B	0.031	0.030	0.031	0.031	0.014
			(0.013)	(0.010)	(0.012)	(0.014)	(0.276)
		C	0.013	0.008	0.000	0.000	−0.960
			(0.012)	(0.008)	(0.001)	(0.000)	(0.196)
		Other	0.045	0.047	0.050	0.050	0.357
			(0.028)	(0.026)	(0.021)	(0.022)	(0.860)
		*Nl*	0.025	0.025	0.025	0.025	−0.011
			(0.003)	(0.002)	(0.002)	(0.004)	(0.082)
		Total	0.114	0.110	0.106	0.106	−0.070
			(0.056)	(0.047)	(0.036)	(0.039)	(0.102)
2	2-strain model	C	0.006	0.002	0.000	0.000	−0.840
			(0.011)	(0.004)	(0.000)	(0.000)	(0.367)
		Other	0.108	0.110	0.108	0.107	−0.007
			(0.011)	(0.009)	(0.009)	(0.009)	(0.058)
		Total	0.114	0.112	0.108	0.107	−0.053
			(0.022)	(0.013)	(0.010)	(0.009)	(0.073)
	4-strain model	B	0.031	0.031	0.031	0.032	0.064
			(0.015)	(0.012)	(0.013)	(0.012)	(0.259)
		C	0.015	0.006	0.000	0.000	−1.00
			(0.014)	(0.006)	(0.001)	(0.000)	(0.000)
		Other	0.047	0.050	0.051	0.052	0.412
			(0.030)	(0.027)	(0.022)	(0.020)	(1.643)
		*Nl*	0.026	0.025	0.025	0.025	−0.014
			(0.003)	(0.002)	(0.003)	(0.003)	(0.078)
		Total	0.118	0.112	0.107	0.108	−0.076
			(0.061)	(0.047)	(0.038)	(0.035)	(0.097)
3	2-strain model	C	0.007	0.001	0.000	0.000	−0.840
			(0.012)	(0.002)	(0.000)	(0.000)	(0.367)
		Other	0.109	0.110	0.108	0.108	−0.007
			(0.011)	(0.030)	(0.010)	(0.010)	(0.060)
		Total	0.116	0.111	0.108	0.108	−0.063
			(0.022)	(0.032)	(0.010)	(0.010)	(0.087)
	4-strain model	B	0.031	0.034	0.032	0.031	0.064
			(0.015)	(0.017)	(0.016)	(0.013)	(0.259)
		C	0.014	0.004	0.000	0.000	−1.00
			(0.010)	(0.007)	(0.001)	(0.000)	(0.000)
		Other	0.046	0.044	0.048	0.052	0.412
			(0.024)	(0.027)	(0.022)	(0.020)	(−0.004)
		*Nl*	0.025	0.025	0.025	0.025	−0.014
			(0.002)	(0.003)	(0.002)	(0.003)	(0.078)
		Total	0.115	0.108	0.105	0.108	−0.061
			(0.052)	(0.054)	(0.040)	(0.036)	(0.103)

Reduction in the prevalence of carriage when an immunization program with the Conjugate C Vaccine is scheduled at 12 month (program 1), 12 years (program 2), and both 12 months and 12 years (program 3) beginning at t = 150 years. Results for both 2-strain and 4-strain models are shown

**Table 4:** Reduction in prevalence of carriage with the MVC-4 vaccine

Vaccine Program	Model Type	Serogroup	t = 149	t = 160	t = 175	t = 190	Relative Change
4	4-strain model	B	0.031	0.031	0.035	0.036	0.202
			(0.014)	(0.013)	(0.013)	(0.010)	(0.259)
		C	0.015	0.011	0.001	0.000	−0.958
			(0.013)	(0.009)	(0.001)	(0.000)	(0.198)
		Other	0.045	0.037	0.011	0.001	−0.974
			(0.026)	(0.022)	(0.010)	(0.003)	(0.052)
		*Nl*	0.025	0.024	0.023	0.022	−0.118
			(0.003)	(0.002)	(0.002)	(0.003)	(0.080)
		Total	0.117	0.102	0.070	0.059	−0.492
			(0.056)	(0.047)	(0.027)	(0.017)	(0.060)
5	4-strain model	B	0.030	0.032	0.035	0.035	0.206
			(0.014)	(0.015)	(0.016)	(0.017)	(0.275)
		C	0.013	0.006	0.000	0.000	−0.920
			(0.015)	(0.008)	(0.002)	(0.000)	(0.271)
		Other	0.046	0.031	0.008	0.000	−0.971
			(0.028)	(0.020)	(0.009)	(0.001)	(0.139)
		*Nl*	0.026	0.024	0.023	0.023	−0.106
			(0.007)	(0.003)	(0.002)	(0.005)	(0.135)
		Total	0.115	0.093	0.067	0.059	−0.487
			(0.064)	(0.046)	(0.029)	(0.023)	(0.087)
6	4-strain model	B	0.029	0.031	0.033	0.034	0.244
			(0.019)	(0.017)	(0.019)	(0.020)	(0.352)
		C	0.016	0.017	0.000	0.000	−0.960
			(0.019)	(0.009)	(0.000)	(0.000)	(0.196)
		Other	0.043	0.021	0.003	0.000	−0.957
			(0.034)	(0.017)	(0.005)	(0.000)	(0.196)
		*Nl*	0.025	0.024	0.022	0.022	−0.121
			(0.004)	(0.002)	(0.003)	(0.002)	(0.066)
		Total	0.113	0.082	0.059	0.057	−0.499
			(0.075)	(0.045)	(0.028)	(0.023)	(0.090)

Reduction in the prevalence of carriage when an immunization program with the quadrivalent MCV-4 vaccine is scheduled for 12 month (program 4), 12 year (program 5), and 12 months and 12 years (program 9) beginning at t = 150 years. Results for the 4-strain model are shown

**Table 5:** Reduction in prevalence of carriage with the 4CMenB vaccine

Vaccine Program	Model Type	Serogroup	t = 149	t = 160	t = 175	t = 190	Relative Change
7	2-strain model	B	0.027	0.021	0.008	0.000	−0.996
			(0.010)	(0.008)	(0.005)	(0.000)	(0.006)
		Other	0.109	0.109	0.107	0.107	−0.017
			(0.009)	(0.007)	(0.007)	(0.009)	(0.056)
		Total	0.136	0.131	0.115	0.107	−0.209
			(0.019)	(0.015)	(0.012)	(0.009)	(0.052)
	4-strain model	B	0.031	0.030	0.022	0.004	−0.861
			(0.014)	(0.023)	(0.015)	(0.014)	(0.000)
		C	0.016	0.016	0.019	0.026	0.665
			(0.014)	(0.013)	(0.015)	(0.021)	(0.007)
		Other	0.046	0.039	0.015	0.028	−0.394
			(0.028)	(0.046)	(0.032)	(0.054)	(0.026)
		*Nl*	0.026	0.025	0.023	0.024	−0.083
			(0.003)	(0.004)	(0.003)	(0.004)	(0.001)
		Total	0.119	0.109	0.079	0.082	−0.308
			(0.059)	(0.086)	(0.066)	(0.093)	(0.240)
8	2-strain model	B	0.027	0.015	0.004	0.000	−0.995
			(0.007)	(0.006)	(0.006)	(0.000)	(−0.006)
		Other	0.110	0.109	0.107	0.108	−0.016
			(0.006)	(0.010)	(0.011)	(0.010)	(0.004)
		Total	0.137	0.124	0.111	0.108	−0.210
			(0.012)	(0.016)	(0.016)	(0.010)	(0.048)
	4-strain model	B	0.033	0.025	0.016	0.003	−0.900
			(0.016)	(0.019)	(0.018)	(0.013)	(0.003)
		C	0.015	0.019	0.023	0.030	0.931
			(0.020)	(0.022)	(0.024)	(0.029)	(0.009)
		Other	0.044	0.021	0.014	0.024	−0.444
			(0.027)	(0.032)	(0.029)	(0.038)	(0.011)
		*Nl*	0.026	0.024	0.023	0.024	−0.075
			(0.002)	(0.004)	(0.003)	(0.003)	(0.001)
		Total	0.117	0.089	0.076	0.081	−0.317
			(0.065)	(0.077)	(0.074)	(0.083)	(0.195)
9	2-strain model	B	0.027	0.013	0.001	0.000	−0.027
			(0.010)	(0.007)	(0.002)	(0.000)	(0.010)
		Other	0.109	0.110	0.107	0.107	−0.999
			(0.010)	(0.008)	(0.008)	(0.009)	(−0.001)
		Total	0.135	0.123	0.108	0.107	−0.206
			(0.020)	(0.015)	(0.011)	(0.009)	(0.057)
	4-strain model	B	0.031	0.025	0.009	0.003	−0.905
			(0.019)	(0.020)	(0.018)	(0.013)	(−0.006)
		C	0.014	0.016	0.023	0.024	0.736
			(0.017)	(0.020)	(0.025)	(0.023)	(0.006)
		Other	0.042	0.022	0.015	0.027	−0.364
			(0.035)	(0.038)	(0.039)	(0.048)	(0.013)
		*Nl*	0.025	0.023	0.023	0.024	−0.060
			(0.003)	(0.003)	(0.004)	(0.024)	(0.002)
		Total	0.112	0.085	0.070	0.078	−0.302
			(0.075)	(0.082)	(0.085)	(0.089)	(0.264)

Reduction in the prevalence of carriage when an immunization program with the 4CMenB vaccine is scheduled for 12 month (program 7), 12 years (program 8), and both 12 months and 12 years (program 9) beginning at t = 150 years. Results for both 2-strain and 4-strain models are shown

### C Vaccine

**Fig. 1:**
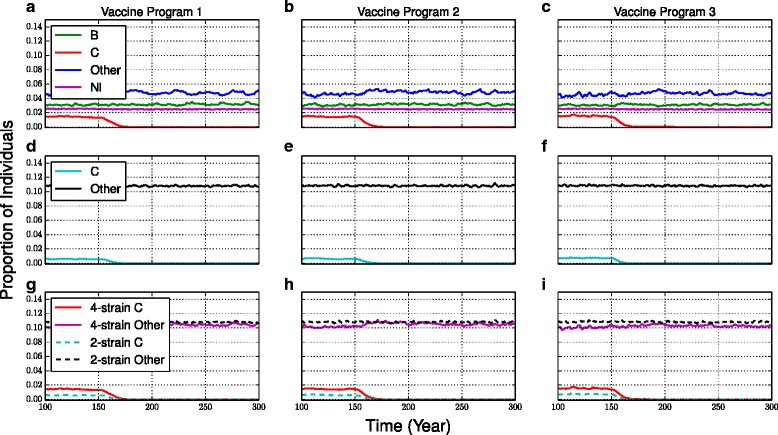
Prevalence of carriage with C vaccine. The prevalence of carriage under vaccine programs 1 (**a**,**d**,**g**), 2 (**b**,**e**,**h**) and 3 (**c**,**f**,**i**) for the 4-strain model (**a**-**c**, showing “C”, “B”, “Nl”, and “Other”), the 2-strain model (**d**-**f**, showing “C” and “Other”), and an overlay of model outputs for both models (**g**-**i**, showing “C” and “Other”). The initial carriage prevalence of serogroup C is different in the 2-strain and 4-strain models because of the filtering procedure used during model parameterization. The same plot with standard deviations of model outputs appears in Additional file 1: Figure S3

In the 4-strain model, introducing the C vaccine causes the prevalence of serogroup C to decline quickly (Fig. 1a-c, see also Additional file 1: Figure S3a-c for time series plots with standard deviation bars on model outputs). The 2-strain model sees a more rapid reduction in the prevalence of carriage for serogroup C (Fig. 1d-f, see also Additional file 1: Figure S3d-f). In the 2-strain model, serogroup C prevalence is reduced to almost zero by approximately t = 165 years, while in the 4-strain model this occurs by approximately t = 170 years. After 15 years, the prevalence of carriage is reduced by 88.1 %, 95.0 %, and 97.3 % for the 4-strain model, compared to 96.3 %, 96.1 %, and 99.9 % for the 2-strain model, for vaccine programs 1–3 respectively (p = 0.0002, 0.0008, and 0.0001, respectively) (Table 3).

The 4-strain model predicts slight strain replacement caused by the C vaccine: the prevalence of “Other” increases slightly while the prevalence of *Nl* and *B* remain constant in vaccine programs 2 and 3, whereas the 2-strain model shows very little evidence of strain replacement, on account of the significant difference in prevalence between serogroup C and “Other”, which includes the very common species *Neisseria lactamica*. This has implications for disease control since different serogroups cause differing pathogenicity.

### MCV-4 Vaccine

**Fig. 2:**
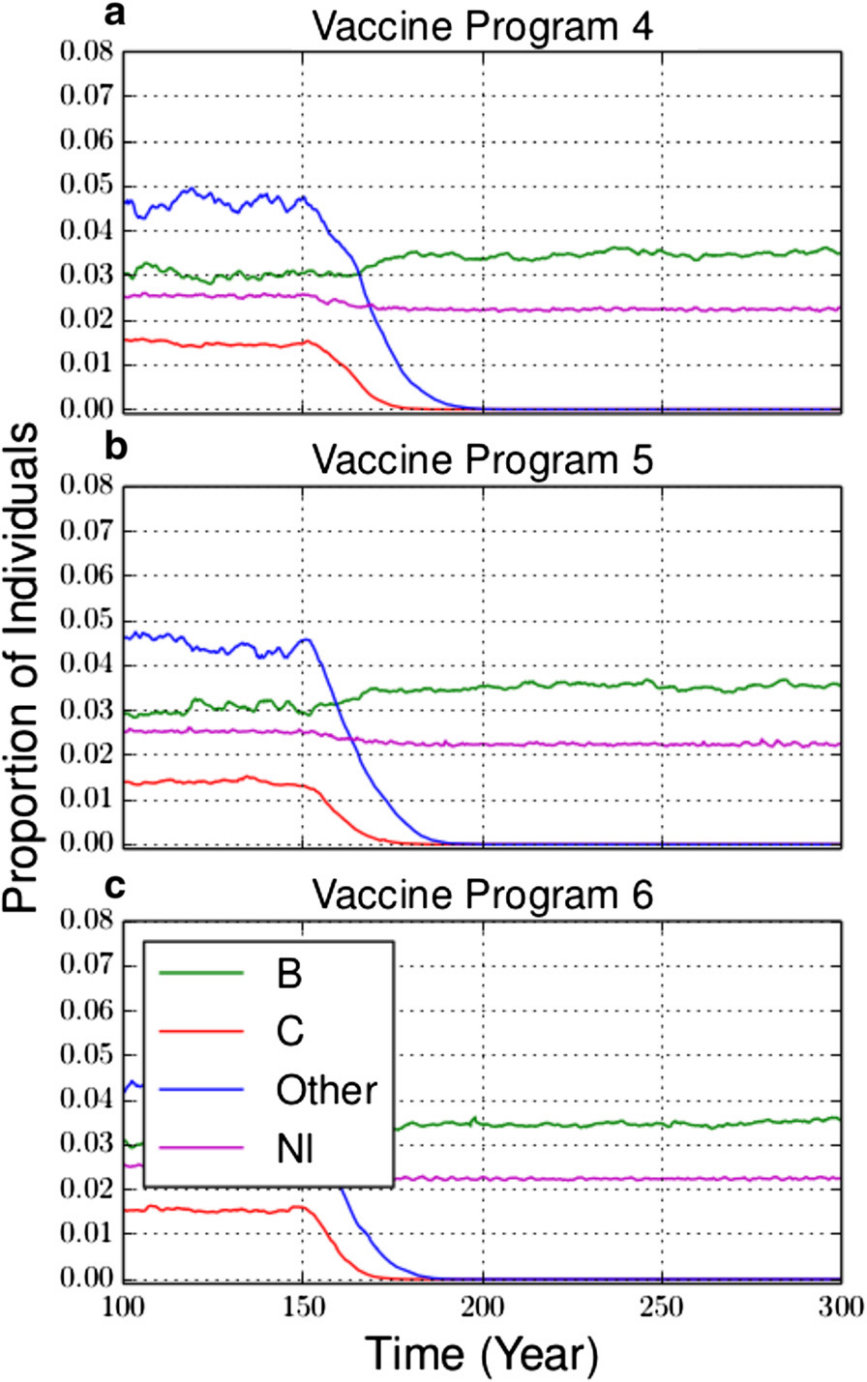
Prevalence of carriage with MCV-4 vaccine. The prevalence of carriage with vaccine program 4 (**a**), 5 (**b**) and 6 (**c**) for the 4-strain model. The same plot with standard deviations of model outputs appears in Additional file 1: Figure S4

Vaccine programs 4 to 6 introduced a quadrivalent ACWY vaccine. In the 4-strain model, this reduced serogroup C as well as the “other” serogroup which partially includes A, W-135 and Y. Similar to the monovalent C vaccine, it takes about 20 years to reduce the prevalence of carriage serogroup C to almost zero. However, it requires an additional 10 years for the “other” serogroup to be reduced to the same level (Fig. 2a-c, see also Additional file 1: Figure S4a-c). The overall reduction in *Nm* prevalence was greater than for the C vaccine, on account of the MCV-4 vaccine including more serogroups. The MCV-4 vaccine causes strain replacement of serogroup B, but not of *Nl,* which is least affected by competition because it has a different duration of carriage than is typical for *Nm* serogroups.

In the 4-strain model, the MCV-4 vaccine programs reduced the overall prevalence of carriage by 49.2 %, 48.7 %, and 49.9 % after 40 years, compared to the reduction of 7.0 %, 7.6 %, and 6.1 % under the C vaccine, for vaccine programs 1–3 respectively (p = <0.0001, <0.0001, and <0.0001, respectively) (Table 4). The reduction in overall prevalence of carriage is higher for the MCV-4 vaccine owing to its greater serogroup coverage than the C vaccine.

### 4CMenB vaccine

**Fig. 3:**
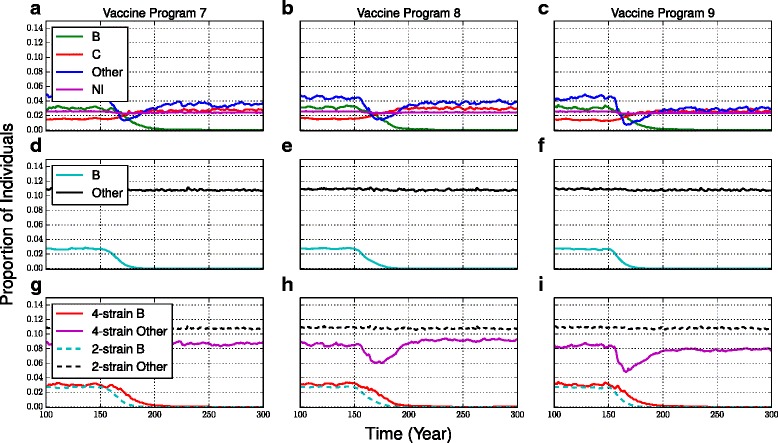
Prevalence of carriage with 4CMenB vaccine. The prevalence of carriage with vaccine programs 7 (**a**,**d**,**g**), 8 (**b**,**e**,**h**) and 9 (**c**,**f**,**i**) for the 4-strain model (**a**-**c**, showing “C”, “B”, “Nl”, and “Other”), the 2-strain model (**d**-**f**, showing “B” and “Other”), and an overlay of model outputs for both models (**g**-**i**, showing “B” and “Other”). The initial carriage prevalence of serogroup “Other” is different in the 2-strain and 4-strain models because of the filtering procedure used during model parameterization. The same plot with standard deviations of model outputs appears in Additional file 1: Figure S5

According to the 4-strain model, 4CMenB vaccine causes the prevalence of carriage to decline at a much slower rate than the decline in serogroups C and “Other” when C and MCV-4 vaccines were introduced. This difference is on account of the lower vaccine efficacy of 4CMenB (Fig. 3a-c, see also Additional file 1: Figure S5a-c). However, as was the case for the C and MCV-4 vaccines, the prevalence of serogroup B drops very close to zero after a sufficient amount of time.

In the 4-strain model, due to the high prevalence of serogroup B, the prevalence of serogroup C increases significantly when the 4CMenB vaccine is introduced on account of strain replacement, growing to a level comparable to that exhibited by serogroup B before the vaccine was introduced to the population (Fig. 3a-c, see also Additional file 1: Figure S5a-c). More interestingly, for vaccine programs 7 and 8, the prevalence of “Other” at first declines along with the prevalence of B, but then rebounds, and for vaccine program 9, the prevalence of “Other” declines steeply and remains low. This suggests that changes in the prevalence of serogroup B may be pushing the prevalence of “Other” to a basin of attraction for an alternative stable state (see next subsection). We also point out that any changes in serogroup C prevalence (caused by vaccine-induced changes to serogroup B prevalence) would also have additional impacts on “Other” serogroup prevalence, and hence the outcomes can be more complicated than would be observed with a fewer-strain model.

The 2-strain model also predicted a slower decline under the 4CMenB vaccine compared to the declines under the C and MCV-4 vaccine (Fig. 3d-f, see also Additional file 1: Figure S5d-f). However, as was observed with the C vaccine, the predicted decline in serogroup B under the 4CMenB vaccine is significantly faster under the 2-strain model than the 4-strain model (see also Fig. 3g-i, Additional file 1: Figure S5g-i). After 30 years of immunization, the 4-strain model predicts a reduction of 65.8 %, 61.7 % and 72.4 % in serogroup B for vaccine programs 7–9 respectively while the 2-strain model predicts a reduction of 93.4 %, 93.7 % and 98.5 % in serogroup B for vaccine programs 7–9 respectively, over the same 30-year time period (p = <0.0001, <0.0001, and <0.0001, respectively) (Table 5). Both 2-strain and 4-strain models predict that vaccinating at 12 months and 12 years of age causes the most rapid declines in prevalence (Table 5).

Unlike the 4-strain model, the 2-strain model predicts no strain replacement, which we again speculate is due to the much higher prevalence of the aggregated “Other” category, which includes *Neisseria lactamica*.

### Apparent multiple attractors

**Fig. 4:**
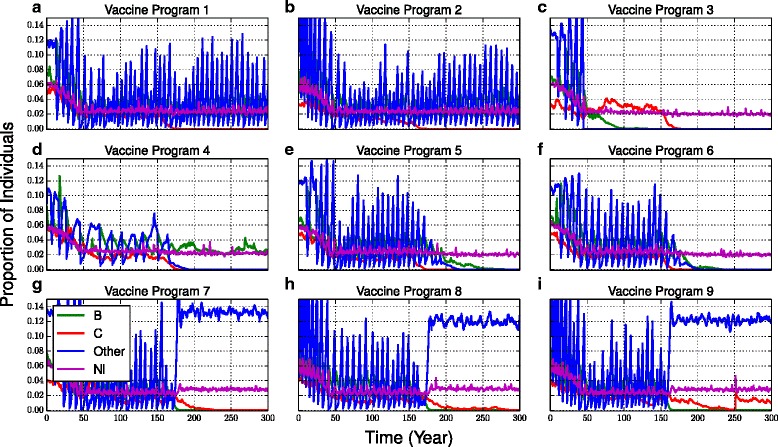
Evidence for multiple attractors in the 4-strain model. Examples of simulations from vaccine scenarios 1–9 (**a**-**i**) that entered the basin of attraction for oscillatory solutions. Some oscillations push serogroup prevalence to zero, causing that serogroup to go extinct in the simulation (no rescue effects were allowed)

During calibration of the 4-strain model it was noted that some parameter sets gave rise to sustained oscillations in serogroup prevalence. These were not included in the foregoing analysis. However, we present a few examples of these dynamics in Fig. 4. Although all serogroups oscillate in prevalence to some extent, the oscillations are particular pronounced for the “Other” serogroup. The C vaccine (programs 1–3, Fig. 4a-c) reduces the prevalence of C without having a significant impact on the oscillations in the other serogroups. The MCV-4 vaccine (programs 4–6, Fig. 4d-f). However, in some simulations (such as the one depicted in Fig. 4d), strain interactions drive serogroup B extinct even before any vaccines are introduced. For the 4CMenB vaccine, the elimination of serogroup B is accompanied by a shift in the dynamics of “Other” from sustained oscillations to a state of high, stable (non-oscillating) prevalence (Fig. 4g-i). The prevalence of C also decreases significantly, probably in response to competition from a surging prevalence of “Other”. The dynamics of a 4-strain model can be rich in ways that cannot be captured with a 2-strain model. We emphasize that these dynamics result from an interaction between forcing the system with the changes induced by the vaccine program, and the dynamical structure of systems with multiple attractors.

## Discussion

Here we developed and compared 2-strain and 4-strain models of the impacts of several types of vaccines on *Neisseria meningitidis* serogroup carriage prevalence. The 2-strain and 4-strain models predicted similar overall impacts of vaccines on carriage prevalence, especially with respect to the vaccine-targeted serogroups. However, declines in vaccine-targeted serogroups were more rapid in the 2-strain model than the 4-strain model, for both the C and the 4CMenB vaccines.

Also, dynamical interactions that are present in the 4-strain model but not the 2-strain model contributed to other differences between the models. Sustained oscillations, and evidence for multiple attractors with differing basins of attraction, occurred in the 4-strain model but not the 2-strain model. In the case of the 4CMenB vaccine, the decline in serogroup B created an ecological niche, but because there are more than two other strains in a 4-strain model, it is not clear *a priori* whether serogroup C or “Other” would step in to fill the niche. In the model simulations, serogorup C was more successful in filling the niche, and the combined effect of changes in serogroup C and B prevalence caused the prevalence of “Other” to at first decrease, and then increase, or simply to decrease and remain low (Fig. 3d-f).

Strain replacement was more pronounced in the 4-strain model than the 2-strain model, we speculate on account of prevalence being more evenly among various serogroups in the 4-strain model, thus enhancing competitive effects. *Neisseria lactamica,* owing to its different natural history, did not experience the competitive effects as strongly as other *Nm* serogroups did from one another.

Oscillations have potentially important impacts on model calibration in multi-strain models. In our case, we excluded parameter sets that yielded oscillations in order to simplify the analysis. However, there are no empirical grounds for excluding such parameter sets, and oscillations in seroprevalence cannot be ruled out based on the existing and rather limited literature reporting cross-sectional results from various populations. Future models should permit oscillatory solutions that satisfy known empirical targets, especially since the qualitative and quantitative impacts of the vaccine could be very different for oscillatory solutions versus steady state solutions.

These types of dynamics appear exotic but they are not exclusive to mathematical models. There is evidence for such dynamics in other infectious disease systems, such as in pertussis before the introduction of vaccines [45]. Unfortunately, because high quality longitudinal data on *Nm* serogroup carriage prevalence do not exist, it is difficult to detect these dynamics in the case of *Neisseria meningitidis.* Moreover, although IMD is a notifiable disease, it is also highly stochastic, which could mask the signs of strain replacement on shorter timescales [2]. Finally, strain replacement effects can be delayed by a number of years, relative to the introduction of a vaccine program that causes them, and hence may not be immediately obvious in IMD case notification time series.

However, as more data on vaccine impacts on IMD incidence and serogroup prevalence become available over time, such new data can be used to further validate dynamic models, which can then be used to evaluate potential expansions of vaccine programs, or applications to other populations that do not currently have vaccine programs. These post-vaccine era data can—and should—be collected so that mathematical models can better inform policy recommendations. There is also value in calibrating the same model to different local circumstances. The additional validation obtained by comparing the model under different epidemiological, vaccine, and demographic circumstances can build confidence in the models so that they may better inform policy. Further work should also explore scenarios using multiple vaccines, since many jurisdictions are currently facing the choice of how best to implement vaccination programs against multiple *Nm* serogroups.

## Conclusions

The phenomena explored in these models may have implications for the predicted effectiveness of *Nm* vaccination. Caution should be exercised when determining which type of model to use to simulate *Neisseria meningitis* dynamics. Models with few strains are easier to analyze, but may miss important features of disease dynamics.

## Additional files

Additional file 1: **Supplementary Material.** Additional file descriptions text (including details of how to view the file, if it is in a non-standard format).

## Abbreviations

Nm: *Neisseria meningitidis*
IMD: Invasive meningococcal disease
MCV-4: Quadrivalent ACWY vaccine
4CMenB: Monovalent B vaccine
Nl: *Neisseria lactamica*
