# Supplementary material for: The impact of aggregating serogroups in dynamic models of Neisseria meningitidis transmission
Source: BMC Infect Dis. 2015 Jul 30;15:300. doi: 10.1186/s12879-015-1015-8 (PMC4520071; doi:10.1186/s12879-015-1015-8)
Supplement: Supplementary file 1 — Supplementary Material. Additional file descriptions text (including details of how to view the file, if it is in a non-standard format). [file 12879_2015_1015_MOESM1_ESM.pdf]

Supplementary Material for

# "The impact of aggregating serogroups in dynamic models of *Neisseria meningitidis* transmission"

Keith D. Poore, Chris T. Bauch

## Table of Contents

**Table S1. All-cause mortality rates by age S3**

Table S2. Parameter values for the 2- and 4-strain models S4

**Table S3. Calibrated $\beta_{i,k}$ values for the 4-strain model S4**

**Table S4. Calibrated $\beta_{i,k}$ values for the 2-strain C vaccine model S5**

**Table S5. Calibrated $\beta_{i,k}$ values for the 2-strain B vaccine model S5**

Table S6. The range of accepted calibration values for the 4-strain model S5

Table S7. The range of accepted calibration values for the 2-strain model analysing the conjugate C Vaccine S6

Table S8. The range of accepted calibration values for the 2-strain model analysing the 4CMenB Vaccine S6

Figure S1. A comparison of the average target and average calibrated prevalence of carriage for the 4-strain model S7

Figure S2. A comparison of the average target and average calibrated prevalence of carriage for the 2-strain models S8

**Figure S3. The prevalence of carriage under vaccine program 1, 2 and 3 for both 4-strain and 2-strain models with error bars showing 2 standard deviations S9**

**Figure S4. The prevalence of carriage under vaccine program 4, 5 and 6 for the 4-strain with error bars showing 2 standard deviations S10**

**Figure S5. The prevalence of carriage under vaccine program 7, 8 and 9 for both 4-strain and 2-strain models with error bars showing 2 standard deviations S11**

*Table 1. All-cause mortality rates (probability of death per year).*

| Age | All-cause Mortality | Age | All-cause Mortality | Age | All-cause Mortality |
|---|---|---|---|---|---|
| <1 | 3.63E-04 | 34 | 6.36E-05 | 68 | 1.25E-03 |
| 1 | 5.18E-05 | 35 | 5.49E-05 | 69 | 1.32E-03 |
| 2 | 2.10E-05 | 36 | 6.85E-05 | 70 | 1.37E-03 |
| 3 | 1.91E-05 | 37 | 9.07E-05 | 71 | 1.55E-03 |
| 4 | 9.26E-06 | 38 | 1.07E-04 | 72 | 1.65E-03 |
| 5 | 8.02E-06 | 39 | 1.18E-04 | 73 | 1.73E-03 |
| 6 | 8.02E-06 | 40 | 1.26E-04 | 74 | 1.86E-03 |
| 7 | 9.87E-06 | 41 | 1.34E-04 | 75 | 2.01E-03 |
| 8 | 7.40E-06 | 42 | 1.89E-04 | 76 | 2.13E-03 |
| 9 | 1.05E-05 | 43 | 1.59E-04 | 77 | 2.21E-03 |
| 10 | 1.36E-05 | 44 | 1.91E-04 | 78 | 2.43E-03 |
| 11 | 9.87E-06 | 45 | 2.18E-04 | 79 | 2.60E-03 |
| 12 | 9.87E-06 | 46 | 2.46E-04 | 80 | 2.88E-03 |
| 13 | 1.60E-05 | 47 | 2.90E-04 | 81 | 2.91E-03 |
| 14 | 1.42E-05 | 48 | 3.22E-04 | 82 | 2.98E-03 |
| 15 | 1.42E-05 | 49 | 3.44E-04 | 83 | 2.96E-03 |
| 16 | 2.28E-05 | 50 | 3.79E-04 | 84 | 3.02E-03 |
| 17 | 2.10E-05 | 51 | 4.13E-04 | 85 | 3.08E-03 |
| 18 | 3.52E-05 | 52 | 4.41E-04 | 86 | 3.08E-03 |
| 19 | 3.02E-05 | 53 | 4.75E-04 | 87 | 3.11E-03 |
| 20 | 4.13E-05 | 54 | 5.18E-04 | 88 | 2.93E-03 |
| 21 | 4.07E-05 | 55 | 5.62E-04 | 89 | 2.75E-03 |
| 22 | 4.20E-05 | 56 | 6.44E-04 | 90 | 2.53E-03 |
| 23 | 3.15E-05 | 57 | 6.55E-04 | 91 | 4.33E-01 |
| 24 | 4.32E-05 | 58 | 7.06E-04 | 92 | 4.33E-01 |
| 25 | 3.64E-05 | 59 | 8.04E-04 | 93 | 4.33E-01 |
| 26 | 3.95E-05 | 60 | 7.94E-04 | 94 | 4.33E-01 |
| 27 | 4.32E-05 | 61 | 8.50E-04 | 95 | 4.33E-01 |
| 28 | 4.26E-05 | 62 | 9.28E-04 | 96 | 4.33E-01 |
| 29 | 5.18E-05 | 63 | 1.09E-03 | 97 | 4.33E-01 |
| 30 | 5.12E-05 | 64 | 1.64E-04 | 98 | 4.33E-01 |
| 31 | 4.50E-05 | 65 | 1.37E-03 | 99 | 4.33E-01 |
| 32 | 6.17E-05 | 66 | 1.19E-03 | | |
| 33 | 5.12E-05 | 67 | 1.26E-03 | | |

*Table S2. Parameters used in the 2-strain and 4-strain models (see Table 1 and 2 in main text for information on vaccine parameters).*

| Parameter | Value | Description |
|---|---|---|
| $\beta_{i,k}$ | calibrated | The age and serogroup specific transmission rate. This value was calibrated [3]. |
| $C_{i,j}$ | age specific | The rate in which individuals from age group *j* come into contact with individuals in age group *i* [40]. Raw data on physical contact rates in the UK from Mossong et al [40] were used, since no Canadian contact data are available. |
| $\sigma_l$ | **0.2** | **Cross immunity conferred against serogroups *m* due to infection by serogroup *l*≠*m* [42]. Cross immunity is assumed to last 24 months for infection by *Nm* and 56.4 months for infection by *Nl* [28, 42].** |
| $\tau_{1,k}$ | 13.0 months for *Nm*; 4.8 months for *Nl* | The average duration of carriage of *Nm* and *Nl* [13, 28]. |
| $\tau_{2,k}$ | 24 months for *Nm*; 56.4 months for *Nl* | The average duration of immunity specific to *Nm* and *Nl* [28, 42]. |
| $\eta$ | 1,300 per month | Number of individuals born per month. This value was assumed. |
| $\mu_i$ | age specific | The death rate of individuals of age *i* in Canada (see Table S1) [39]. |

***Table S3. Average (2 standard deviations) $\beta_{j,k}$ values calibrated for the 4-strain model stratified by age and serogroup.***

| | **<1-4** | **5-9** | **10-14** | **15-19** | **20+** |
|---|---|---|---|---|---|
| **B** | **0.00109 (0.00012)** | **0.00049 (0.00005)** | **0.00065 (0.00014)** | **0.00085 (0.00010)** | **0.00111 (0.00005)** |
| **C** | **0.00084 (0.00079)** | **0.00068 (0.00032)** | **0.00072 (0.00033)** | **0.00097 (0.00021)** | **0.00097 (0.00027)** |
| **Other** | **0.00102 (0.00010)** | **0.00051 (0.00037)** | **0.00053 (0.00052)** | **0.00110 (0.00020)** | **0.00117 (0.00006)** |
| ***N lactamica*** | **0.03898 (0.00240)** | **0.17541 (0.05021)** | **0.01901 (0.00237)** | **0.00532 (0.00020)** | **0.00200 (0.00008)** |

***Table S4. Average (2 standard deviations) $\beta_{j,k}$ values calibrated for the 2-strain C vaccine model stratified by age and serogroup.***

| | <1-4 | 5-9 | 10-14 | 15-19 | 20+ |
|---|---|---|---|---|---|
| **C** | **0.00142 (0.00092)** | **0.00182 (0.00218)** | **0.00161 (0.00120)** | **0.00105 (0.00017)** | **0.00110 (0.00030)** |
| **Other** | **0.00182 (0.00092)** | **0.00278 (0.00214)** | **0.00216 (0.00112)** | **0.00113 (0.00016)** | **0.00124 (0.00030)** |

***Table S5. Average (2 standard deviations) $\beta_{j,k}$ values for the 2-strain B vaccine model stratified by age and serogroup.***

| | <1-4 | 5-9 | 10-14 | 15-19 | 20+ |
|---|---|---|---|---|---|
| **B** | **0.00117 (0.00010)** | **0.00089 (0.00005)** | **0.00096 (0.00004)** | **0.00104 (0.00006)** | **0.00115 (0.00001)** |
| **Other** | **0.00211 (0.00000)** | **0.00348 (0.00000)** | **0.00252 (0.00000)** | **0.00118 (0.00000)** | **0.00133 (0.00000)** |

*Table S6. The range of acceptability for prevalence of carriage during calibration for each serogroup and age group for the 4-strain model.*

| | B | C | Other | NI |
|---|---|---|---|---|
| <1 - 4 | (0.006, 0.028) | (0.000, 0.021) | (0.008, 0.061) | (0.087, 0.154) |
| 5-9 | (0.009, 0.034) | (0.001, 0.025) | (0.012, 0.072) | (0.073, 0.166) |
| 10-14 | (0.012, 0.040) | (0.000, 0.026) | (0.017, 0.083) | (0.053, 0.109) |
| 15-19 | (0.026, 0.063) | (0.009, 0.043) | (0.043, 0.128) | (0.025, 0.063) |
| 20+ | (0.024, 0.032) | (0.012, 0.020) | (0.044, 0.062) | (0.010, 0.039) |

*Table S7. The range of acceptability for prevalence of carriage during calibration for each serogroup and age group for the 2-strain model used to analyse the C vaccine predictions.*

| | C | Other |
|---|---|---|
| <1 - 4 | (0.000, 0.021) | (0.102, 0.238) |
| 5-9 | (0.001, 0.025) | (0.094, 0.267) |
| 10-14 | (0.000, 0.026) | (0.081, 0.225) |
| 15-19 | (0.009, 0.043) | (0.092, 0.245) |
| 20+ | (0.012, 0.020) | (0.075, 0.129) |

*Table S8. The range of acceptability for prevalence of carriage during calibration for each serogroup and age group for the 2-strain model used to analyse the 4CMenB vaccine predictions.*

| 2-strain model for modelling 4CMenB vaccine | | |
|---|---|---|
| | B | Other |
| <1 - 4 | (0.006, 0.028) | (0.095, 0.215) |
| 5-9 | (0.009, 0.034) | (0.085, 0.238) |
| 10-14 | (0.012, 0.040) | (0.070, 0.191) |
| 15-19 | (0.026, 0.063) | (0.068, 0.191) |
| 20+ | (0.024, 0.032) | (0.054, 0.100) |

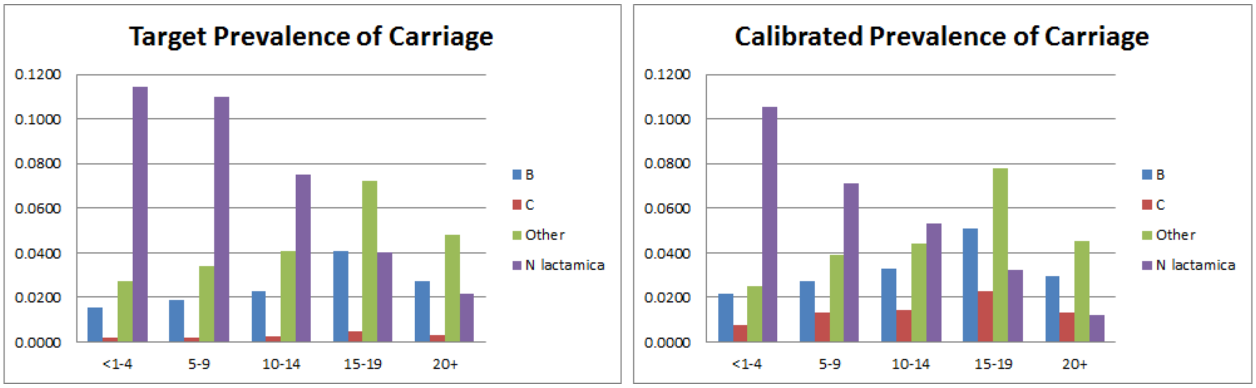


*Figure S1. Calibration targets and model-calibrated prevalence of carriage. Fadeout occurred too frequently for serogroup C at the empirical carriage level. This created a computational limitation hence, the model target was chosen to be the smallest value at which the fadeout did not occur, which is approximately 5 times greater than in [3]. The target prevalence was set from the epidemiological literature [3]. The calibrated prevalence is the average resulting prevalence after the model has been calibrated.*

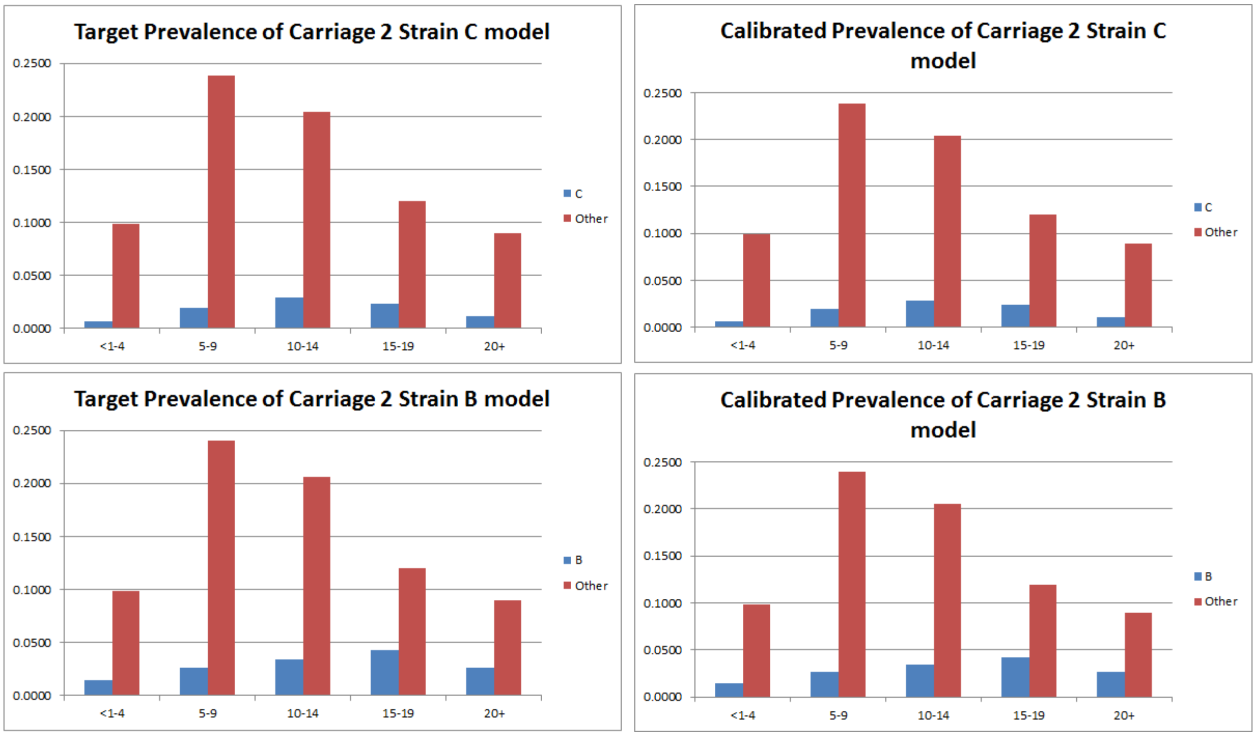


*Figure S2 Calibration targets and model prevalence of carriage for the 2-strain model. Fadeout occurred frequently with serogroup C at the empirical carriage level. This created the same limitations stated in Figure S1. The target prevalence was set from the epidemiological literature [3]. The calibrated prevalence is the average resulting prevalence after the model has been calibrated.*

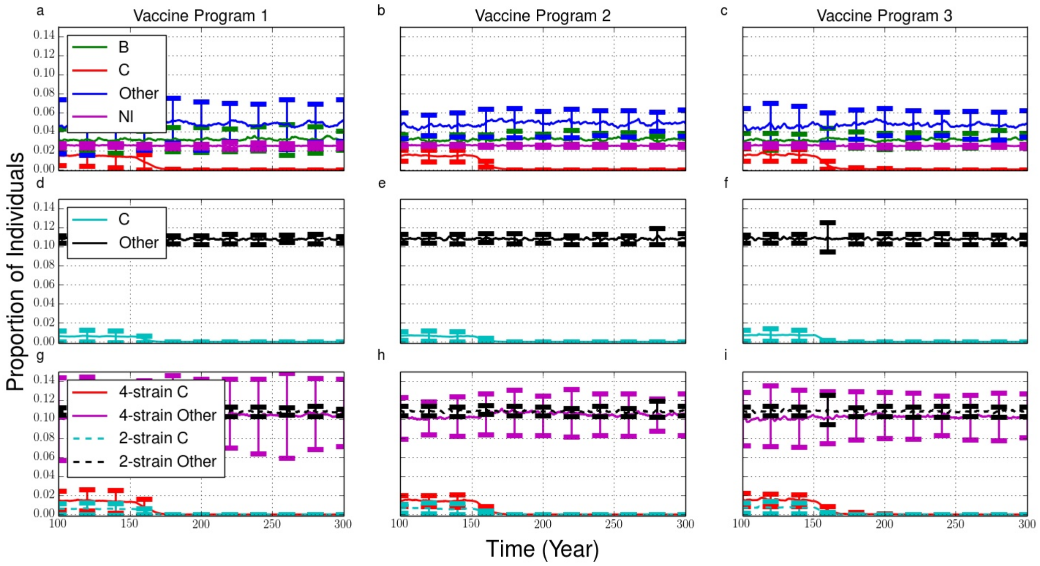


**Figure S3. The prevalence of carriage under vaccine program 1 (a,d,g), 2(b,e,h) and 3(c,f,i) for both 4-strain (a-c) and 2-strain (d-f) models with error bars showing 2 standard deviations, and an overlay of model outputs for both models (g-i, showing "C" and "Other").**

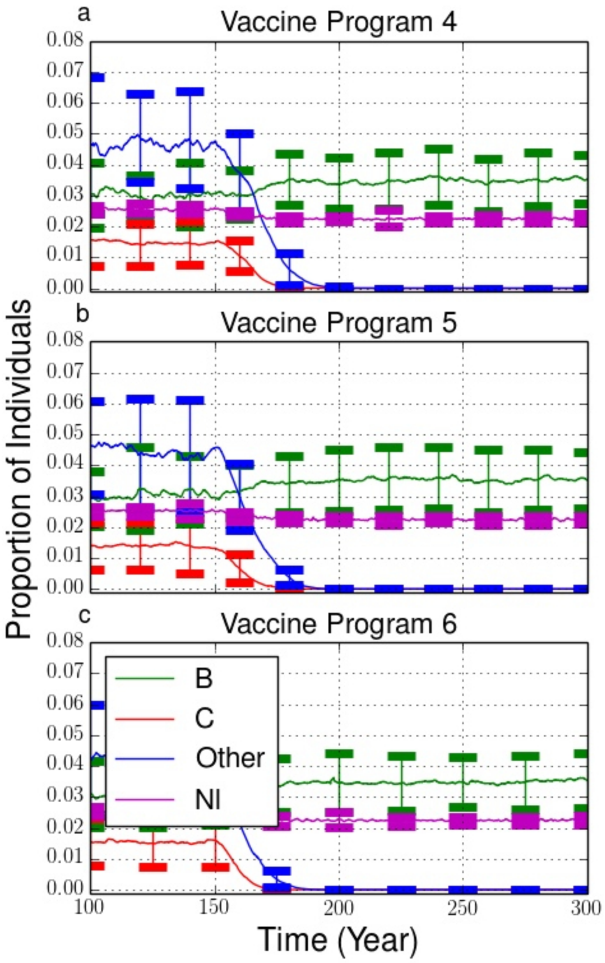


**Figure S4. The prevalence of carriage under vaccine program 4 (a), 5(b) and 6(c) for the 4-strain (a-c) model with error bars showing 2 standard deviations.**

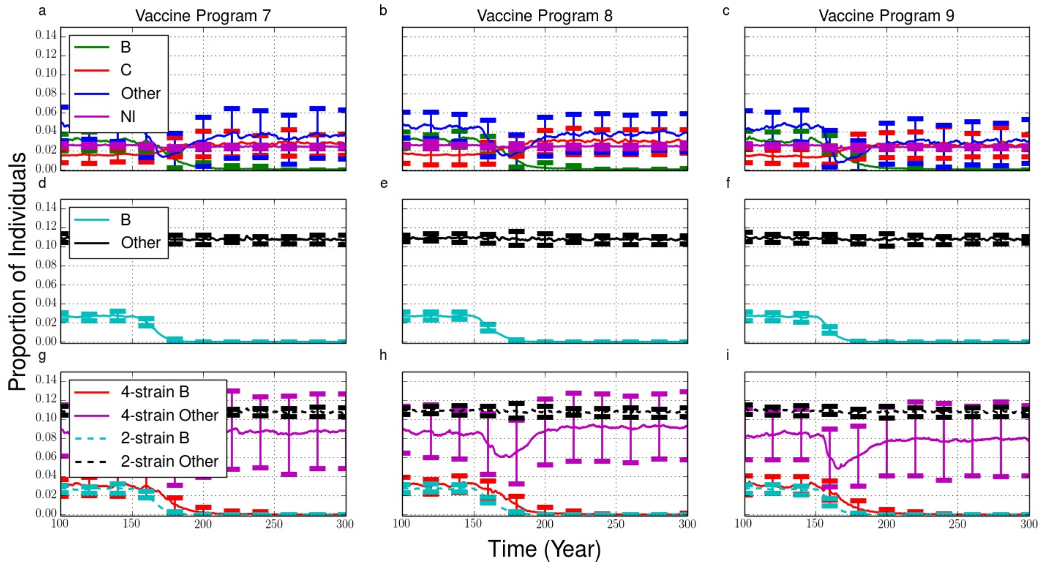


**Figure S5. The prevalence of carriage under vaccine program 7 (a,d,g), 8(b,e,h) and 9(c,f,i) for both 4-strain (a-c) and 2-strain (d-f) models with error bars showing 2 standard deviations, and an overlay of model outputs for both models (g-i, showing "B" and "Other").**
